# MIG (CXCL9) is a more sensitive measure than IFN-γ of vaccine induced T-cell responses in volunteers receiving investigated malaria vaccines

**DOI:** 10.1016/j.jim.2008.09.021

**Published:** 2009-01-01

**Authors:** Tamara K. Berthoud, Susanna J. Dunachie, Stephen Todryk, Adrian V.S. Hill, Helen A. Fletcher

**Affiliations:** a University of Oxford, Centre for Clinical Vaccinology and Tropical Medicine, Churchill Hospital, Oxford, OX3 7LJ, UK; b School of Applied Sciences, Northumbria University, Newcastle-upon-Tyne, UK

**Keywords:** 7AAD, 7-amino-actinomycin D, AMA1, apical membrane antigen 1, APC, antigen presenting cell, BME, β-mercaptoethanol, CFP10, culture filtrate protein 10, CMV, cytomegalovirus, CSP, circumsporozoite protein, CXCL9, CXC chemokine ligand 9, DOC, day of challenge, EBV, Epstein Barr Virus, ELISA, enzyme linked immunoSorbant assay, ELISPOT, enzyme linked immuno SPOT, ESAT6, six-kDa early secreted antigenic target, F, FP9, FP9, fowlpox strain FP9, HPRT, hypoxanthine phosphoribosyl transferase, IFN-γ, interferon gamma, ICS, intracellular staining, JAK, janus kinase, M, MVA, ME, multi-epitope string, MIG, monokine induced by gamma, MVA, modified vaccinia virus Ankara, PBMC, peripheral blood mononuclear cells, P, PEV3A, PHA, phytohaemagglutinin, PPD, purified protein derivative, RPMI, Roswell Park Memorial Institute media, RLT, RNA lysis buffer, RT-PCR, reverse transcription polymerase chain reaction, SEB, staphylococcal enterotoxin B, STAT, signal transducers and activators of transcription, TRAP, thrombospondin related adhesive protein., Malaria, MIG, CXCL9, Vaccine, IFN-γ, Flow cytometry

## Abstract

For many years the IFN-γ *ex vivo* ELISPOT has been a major assay for assessing human T-cell responses generated by malaria vaccines. The ELISPOT assay is a sensitive assay, but an imperfect correlate of protection against malaria. Monokine induced by gamma (MIG), or CXCL9, is a chemokine induced by IFN-γ and has the potential to provide amplification of the IFN-γ signal. MIG secretion could provide a measure of bio-active IFN-γ and a functional IFN-γ signalling pathway. We report that detecting MIG by flow cytometry and by RT-PCR can be more sensitive than the detection of IFN-γ using these methods. We also find that there is little inter-individual variability in MIG secretion when detected by flow cytometry and that the MIG assay may be used to estimate the amount of bio-active IFN-γ present. Measurement of MIG alongside IFN-γ may provide a fuller picture of Th1 type responses post-vaccination.

## 1 Introduction

Malaria kills over one million people every year, the majority of whom are children (Breman et al., 2001). Vaccination may offer one way of effectively treating or even eradicating this disease.

Research into the pre-erythrocytic stage of the malaria parasite's life cycle has shown that many factors may be involved in protection from malaria in humans. Antibodies, T-cells and cytokines, particularly IFN-γ, have all been shown to associate with protection against malaria, in both mice (García-López, 2001; Pinder et al., 2004; Vuola et al., 2005) and humans (Schofield et al., 1987; Weiss et al., 1988; Renia et al., 1991; Nussler et al., 1993; Khusmith et al., 1994; Reece et al., 2004). IFN-γ measured by the *ex vivo* IFN-γ ELISPOT has been the primary readout of immunogenicity for T-cell inducing vaccines in clinical trials (Vuola et al., 2005; Bejon et al., 2006; Dunachie et al., 2006). The *ex vivo* ELISPOT assay enables determination of the number of IFN-γ secreting cells but not the quantity of IFN-γ secreted or the functionality of the cytokine. In general, the vaccine regimens which induce high IFN-γ *ex vivo* ELISPOT responses have been shown to induce greater protection (Webster et al., 2005)and following vectored vaccines, the IFN-γ *ex vivo* ELISPOT has been found to correlate with protection from malaria (Dunachie et al., 2006). Whilst IFN-γ is involved in protection in some malaria models (Meding et al., 1990; Stevenson et al., 1990; Bruna-Romero et al., 2001), it is likely that other factors are involved in human malaria and the *ex vivo* IFN-γ ELISPOT assay is not able to clearly measure all the protective features of the immune response to malaria (Flanagan et al., 2003; John et al., 2004). An alternative measure may be more sensitive and may correlate better with protection from malaria.

The secretion of monokine induced by gamma (MIG: CXC chemokine ligand 9 (CXCL9) has been examined as an alternative marker of immunogenicity (Brahmbhatt et al., 2002).

MIG, a member of the CXC subfamily of chemokines is an inflammatory chemokine that is important in the recruitment of activated T-cells to sites of infection. MIG enhances Th1 and Th2 polarization, attracting Th1 cells and inhibiting Th2 migration. A strong MIG mediated Th1 response has been shown to be important in protection from *Trypanosoma cruzi* (Hardison et al., 2006). There are two features of the MIG response that make it an attractive chemokine to measure. Firstly, MIG is induced by IFN-γ, which may allow it to provide a functional measure of IFN-γ activity (Farber, 1993). Secondly MIG is produced following an amplification of the IFN-γ signal and may be easier to detect and a more sensitive measure of bio-active IFN-γ than detecting IFN-γ directly (Brice et al., 2001).

MIG is secreted by macrophages, monocytes, neutrophils, APC, B cells and eosinophils (Whiting et al., 2004). However, CD14+ cells which include monocytes and macrophages are thought to comprise the majority of MIG secreting cells (Loetscher et al., 1996; Brice et al., 2001). MIG secretion by these cells is induced by IFN-γ and mediated via the JAK-STAT signalling pathway (Sarkar et al., 2007). MIG secretion is therefore an indication of a functional JAK-STAT signal from the IFN-γ receptor. It has also been reported that MIG can be induced by IFN-α in IFN-γ^−/−^ mice, when IFN-α and IFN-γ are both inhibited, MIG expression is completely absent (Mahalingam et al., 2001).

Antigen specific MIG secretion, detected by ELISA and flow cytometry, in two studies have reported MIG detection to be a sensitive measure of immunogenicity (Brice et al., 2001; Abramo et al., 2006). Brice et al. were able to demonstrate the detection of malaria peptide specific MIG production in one subject vaccinated with irradiated sporozoites. Our study of 12 subjects is the first evaluation of MIG and IFN-γ detection by flow cytometry in the context of a phase I vaccine trial of a recombinant viral vector vaccine. This study aimed to test the ability of MIG as a sensitive marker of viral vectored recombinant malaria vaccine that induces immunogenicity. The aforementioned studies did not compare both MIG and interferon gamma production directly using intracellular staining. Therefore this study directly compared the sensitivity of MIG and IFN-γ by intracellular staining and RT-PCR in the context of recombinant malaria vaccines.

## 2 Methods

### 2.1 Subjects and vaccine regimens

Volunteers recruited for this malaria vaccine trial were malaria-naive, male or female Caucasians aged 18–65 years. Volunteers received six separate vaccinations administered in three visits as described elsewhere (Thompson et al., 2008). The first and second immunisations consisted of one dose of the viral vaccine fowlpox strain 9 (FP9) encoding thrombospondin adhesive protein with a multi-epitope string (ME-TRAP) (1 × 10^8^ pfu) and one dose of the virosomal vaccine PEV3A that includes cyclised peptides from the circumsporozoite protein (CSP) and the apical membrane antigen 1 (AMA1) of *Plasmodium falciparum*. PEV3A was given as 0.5 ml containing 50 mg of PEV301 (AMA-49) and 10 mg of PEV302 (UK39) in phosphate buffered saline (pH 7.4). The third immunisation consisted of PEV3A given in conjunction with one dose of the viral vaccine Modified Vaccinia virus Ankara (MVA) encoding ME-TRAP (1.5 × 10^8^ pfu). All the immunisations were given four weeks apart. The vaccine regimen will be referred to as PFPFPM, P representing PEV3A, F representing FP9 encoding ME-TRAP, and M representing MVA encoding ME-TRAP. Blood was analysed for CS and TRAP specific immune responses before any vaccine was administered (D0), seven days after the final vaccination (V3 + 7) and on the day of malaria challenge (DOC), which was 14 days after the final vaccination (V3 + 14). Further details of this trial are reported elsewhere (Thompson et al., 2008).

Peripheral blood mononuclear cells (PBMC) were isolated from buffy coats from healthy blood donors (National Blood Service, Bristol) and used for the optimisation experiments.

### 2.2 PBMC separation

PBMC were separated using a density gradient. Blood was collected from each volunteer into a heparin tube and processed within 6 h of collection. Fifteen to thirty milliliters of blood was layered onto 15 ml of lymphoprep (Axis-Shield, Oslo, Norway) pre-aliquoted into leucosep tubes (Greiner-Bio-One Laboratories, Friekenhausen, Germany). The leucosep tubes were centrifuged and PBMC collected from just above the lymphoprep. PBMC were washed twice with Roswell park memorial institute media (RPMI) and counted before use in the assays. PBMC obtained from the Blood Donation Service (Bristol UK) were separated as described above following 1/2 dilution with RPMI.

### 2.3 Stimulant peptides

One pool containing 57 × 20 mer peptides spanning the whole of the TRAP protein was used at a concentration of 10 µg/ml. Another pool containing 23 × 9 mer CD8+ T-cell viral epitopes from Cytomegalovirus (CMV), Epstein Barr Virus (EBV), and influenza, was used at a concentration of 10 µg/ml (Good et al., 1993). These will be referred to as the CEF pool. Purified protein derivative (PPD) was used at a concentration of 10 µg/ml. Phytohaemagglutinin (PHA) and Staphylococcal enterotoxin B (SEB) were used as positive controls at a concentration of 1 µg/ml.

### 2.4 *Ex vivo* IFN-γ ELISPOT assay

Fifty micro-liters of isolated PBMC (0.4 × 10^6^ cells) and 50 µl of stimulant peptide were added to an ELISPOT plate (MultiScreen-IP plates; Millipore, Watford, United Kingdom) pre-coated the day before with 50 µl of IFN-γ capture antibody (1-D1K; MabTech, Nacka, Sweden) at 10 µg/ml in carbonate buffer (Sigma, Poole, UK) then blocked with 100 µl of R10 (R10: RPMI 1640 with 10% fetal calf serum, 100 IU/ml penicillin, 0.1 mg/ml streptomycin (all Sigma, Poole, UK), and 2 mM l-glutamine (GIBCO/Invitrogen, Paisley, UK)). The plates were incubated for 18–20 h at 37 °C. After washing six times with PBS-Tween (Sigma, Poole, UK), 50 µl of 1 µg/ml of detector antibody (7-B6-1-Biotin Mabtech, Nacka, Sweden) was added. Fifty micro-liters of 1/1000 Streptavidin conjugated with enzyme —ALP (Mabtech, Nacka, Sweden) was added following another wash with PBS-Tween (Sigma, Poole, UK). The plates were developed with a precipitating substrate ALP kit (Bio-rad, Hercules, CA) according to the manufacturer's instructions. The plates were dried and read using an AID ELISPOT reader (AID, Strassberg, Germany).

### 2.5 Intracellular staining (ICS)

Cryopreserved PBMC were thawed quickly and added to 1 ml of R10 with 25 U/ml benzonase nuclease (Novagen). Cells were then diluted with a further 9 ml of R10, washed once and resuspended for counting (CasyCounter TT Schärfe System, Reutlingen, Germany). Either 100 µl of 1 × 10^6^ PBMC, or 50 µl of 1 × 10^6^ PBMC with 50 µl of 0.1 µg/ml anti-CD28 and anti-CD49d antibodies (BD Pharmingen, Oxford, UK) were cultured for MIG ICS analysis. Two million PBMC in 50 µl with 50 µl of 0.1 µg/ml anti-CD28 and anti-CD49d antibodies were cultured for IFN-γ ICS analysis. Fifty micro-liters of stimulant peptides were added to both assays. Unless otherwise stated the cells were then incubated for 6 h at 37 °C before the addition of 0.1 µg/ml Brefeldin A (BD Pharmingen, Oxford, UK). The cells were then incubated at 37 °C for 6 h unless otherwise stated.

Stains used were MIG ICS (CD14-FITC — clone M5E2), IFN-γ ICS (CD3-PE-cy7 clone SK7), CD4 (APC-cy7 clone RPA-T4) and CD8 (FITC clone SK1). 7AAD was used as a dead cell stain in both the MIG and IFN-γ analysis.

Cells were analysed using a FACSCalibur flow cytometer (FACSCalibur, BD Biosciences). The MIG data is presented as the percentage of MIG positive cells within the CD14+, 7AAD-ve gate. Thirty thousand to fifty thousand events were collected, which ensured at least 1000 events in the MIG+ CD14+ 7AAD-ve gates in the positive controls were collected. The IFN-γ data is presented as the percentage of IFN-γ+ cells within the CD3+ 7AAD-ve gate. Seven hundred thousand events were collected which ensured that at least 600 events were collected in the IFN-γ+ CD3+ 7AAD-ve gates in the positive controls. For the vaccine trial subjects the data is presented as the number of cytokine (MIG or IFN-γ) positive cells in the TRAP stimulated samples minus the number of cytokine positive cells in the unstimulated media alone sample. A positive result was determined by the sample being over 3 standard deviations above the mean of the media wells at the baseline time point.

### 2.6 Real-time RT-PCR

RNA extraction was carried out using the RNeasy Mini-kit (Qiagen, Crawley, UK). Extractions were carried out according to the manufacture's recommendations following a 12 h incubation with peptides. Twelve hours has been reported to detect early, mid and late gene expression in response to antigen stimulation (Huang et al., 2001). Briefly, cells were lysed in 100 µl RNA Lysis Buffer (RLT) containing 10% β-mercaptoethanol (BME). One hundred micro-litres of 70% ethanol was added to each sample, which was then applied to an RNeasy mini column. The columns were then washed 3 times and RNA eluted into 30 µl of RNase free water.

RNA was reverse transcribed to cDNA using Omniscript reverse transcriptase (Omniscript kit, Qiagen, Crawley, UK) with oligo-dT-primers (MWG Biotech, Milton Keynes, UK) according to the manufacture's instructions.

Real time PCR was carried out using a Light Cycler (Roche). PCR master mix consisted of 10 µl Quantitect (Qiagen Crawley, UK) and 10 pmol of each primer (Forward and Reverse) diluted with water. Nineteen micro-litres of master mix and 1 µl of cDNA template was added to each light cycler tube.

Primers were used:

HPRT (F 5′-TATGGACAGGACTGAACGTC-3′ and R 5′-CTACAATGTGATGGCCTCCC-3′), MIG (F-5′GCATCATCTTGCTGGTTCTGATTGG-3′, and R 5′-GCGACCCTTTCTCACTACTGGGGT-3′), IFN-γ (F-5′ AGCCATCTCTGTCCTC-3′ and R 5′-TTCTGCTCTGACAACCT-3′), FOXP3 (F-5′ CACTTACAGGCACTCCTCCAGG and R5′-CCACCGTTGAGAGCTGGTGCAT), IFN-α (F 5′-AGCCATCTCTGTCCTC-3′ and R 5′-TTCTGCTCTGACAACCT-3′). PCR amplification conditions were: 94 °C—15 min, followed by 50 cycles of 94 °C—15 s, 60 °C—15 s and 72 °C—15 s.

The results are presented as the mRNA fold change of the TRAP stimulated samples against the unstimulated media alone samples.

### 2.7 Statistical analysis

IFN-γ, MIG and FoxP3 expression were normalised by dividing copy number of gene by copy number of the house keeping gene hypoxanthine phosphoribosyl transferase (HPRT). Paired Student's *t*-test was used to analyse normally distributed data and Wilcoxon rank sum or a Mann–Whitney *U* test was performed on non-normally distributed data. Spearman's test was used for correlation. The statistics were calculated using SPSS for Windows Version 12.0.

## 3 Results

### 3.1 The increase in MIG mRNA expression is greater than the increase in IFN-γ mRNA expression

**Fig. 1:**
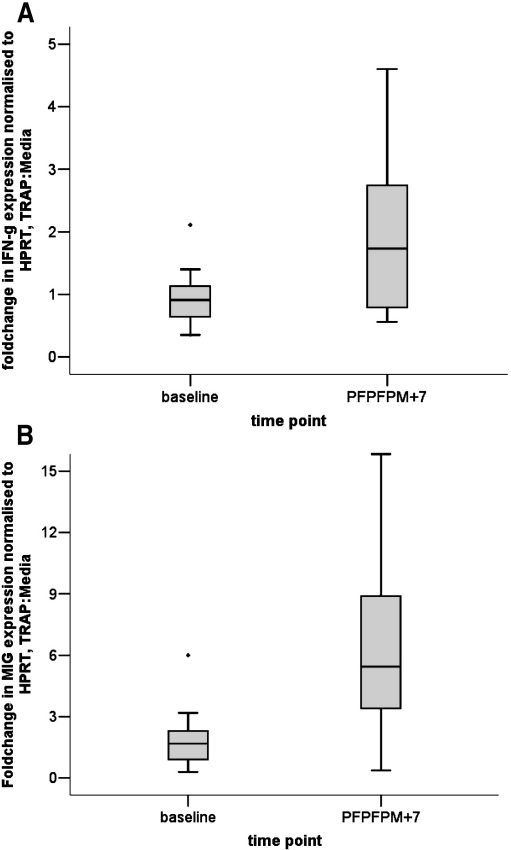
(A) shows box and whisker plot of the MIG mRNA fold change; TRAP stimulated against the media alone. The samples were tested at the pre-vaccination time-point D0, and 7 days after the final vaccination V3+7 (n=10). The samples were incubated for 12 hours before mRNA was extracted. A 5.2 fold change was seen from D0 to the V3+7 time point. (B) shows a box and whisker plot of IFN-γ mRNA fold change; TRAP stimulated against the media alone samples, at the pre vaccination time-point D0 and 7 days after the final vaccination V3+7. A fold change of 1.7 was seen from D0 to the V3+7 time point. A significant difference was seen between the fold change in IFN-γ expression and the fold change in MIG expression (Wilcoxon Signed Ranks Test P = 0.013).

PBMC from 10 volunteers vaccinated with the PFPFPM regimen, were stimulated with TRAP peptides. Real-time RT-PCR was used to quantify MIG and IFN-γ expression in the stimulated and unstimulated cells from the pre-vaccination time point (D0) and 7 days after the final vaccination (V3 + 7). The expression of IFN-γ and MIG were normalised to HPRT. The median fold increase; TRAP stimulated against the media alone, in IFN-γ expression at the V3 + 7 time point was 1.7 (Fig. 1A) compared to a median fold increase at the V3 + 7 time point of 5.2 in MIG expression (Fig. 1B). A significant difference was seen between the fold change in IFN-γ expression and the fold change in MIG expression (Wilcoxon Signed Ranks Test *P* = 0.013) at this time point.

### 3.2 Optimisation of incubation time, before the addition of brefeldin A for the MIG and IFN-γ ICS assays

MIG is secreted following binding of IFN-γ to its receptor in monocytes and macrophages. The interferon signal is amplified in monocytes and macrophages, and MIG mRNA is produced at a higher level than IFN-γ mRNA (Fig. 1).

**Fig. 2:**
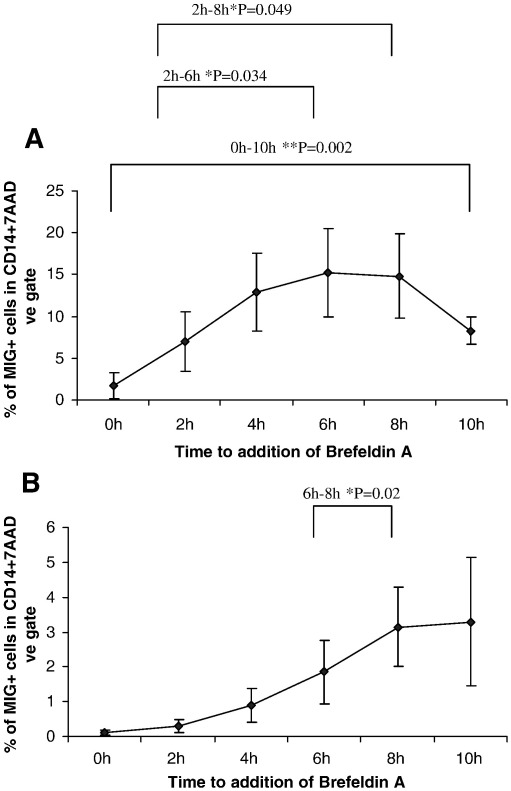

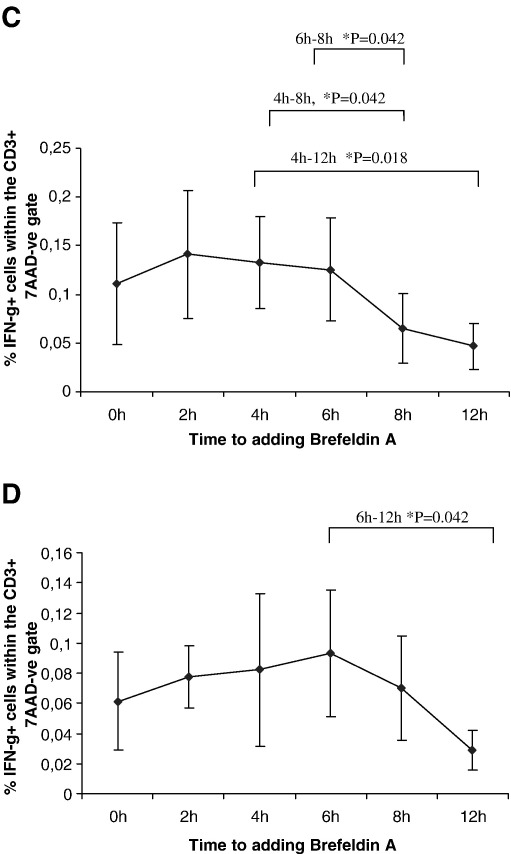
(A) MIG ICS response to CEF peptides. (B) MIG ICS response to PPD. (C) IFN-γ ICS responses to CEF peptides. (D) IFN-γ ICS responses to PPD. Optimization of the incubation time with CD8+ viral epitopes (CEF) (panels A and C) and PPD (panels B and D) before addition of brefeldin A. Brefeldin A was added at 2 hourly intervals after addition the antigens. Samples were analysed after a further 18 h of incubation. The data is presented as the mean percentage of MIG+ cells ± S.E (panels A and B) (*n* = 5) and the mean percentage of IFN-γ+ cells ± S.E (panels C and D) (*n* = 9), with background media only wells deleted. A significant difference in the CEF stimulated samples (panel A) was seen between 0 and 10 h, 2 and 6 h, and 2 and 8 h (*P* = 0.002 for 0 and 10 h, *P* = 0.034 for 2 and 6 h, *P* = 0.049 for 2 and 8 h, Student's paired *T*-test). A significant difference in the PPD stimulated samples (panel B) was seen between 6 and 8 h (*P* = 0.02 Student's paired *T*-test). A significant difference in IFN-γ productions following CEF stimulation (panel C) was seen between 4 and 8 h, 6 and 8 h, and 4 and 12 h (*P* = 0.042 for 4 and 8 h, *P* = 0.042 for 6 and 8 h, *P* = 0.042 for 4 and 12 h, Wilcoxon signed rank test). A significant difference in IFN-γ production was seen in the PPD stimulated samples (panel D) was seen between 6 and 12 h (*P* = 0.042 Wilcoxon signed rank test).

Brefeldin A blocks proteins from being secreted. Brefeldin A may therefore prevent IFN-γ from being secreted and thus from inducing MIG. PBMC from 5 normal human donors were stimulated with either a mixture of viral CD8+ T-cell epitopes (CEF) (Fig. 2A) or PPD (Fig. 2B). The cells were incubated for 0, 2, 4, 6, 8 and 10 h before brefeldin A was added, and incubated for a further 18 h after brefeldin A was added.

A longer incubation time before the addition of brefeldin A resulted in the detection of a higher number of MIG secreting CD14+ cells. A significantly higher percentage of CD14+ MIG+ cells were detected at 6 h and 8 h compared to 2 h (Student's paired *t*-test *P* = 0.034 for 2 and 6 h, *P* = 0.049 for 2 and 8 h ) in the CEF stimulated cells. A significant increase was also seen from 6 h to 8 h, (Student's paired *t*-test *P* = 0.02) in the PPD stimulated cells.

Optimisation tests were also carried out to determine the optimal incubation time for detecting IFN-γ. PBMC from 9 normal human donors were selected and stimulated with CEF (Fig. 2C) or PPD (Fig. 2D). No significant differences were detected between the early time points with either stimulation (0 h–6 h). However a significant decrease in the percentage of IFN-γ secreting cells was detected between 4 and 8 h (*P* = 0.042) and 4 and 12 h (*P* = 0.018), as well as between 6 and 8 h (*P* = 0.042) (Wilcoxon rank sum test). Following PPD stimulation, a significant drop in the percentage of IFN-γ was detected between 6 and 12 h (P = 0.042 Wilcoxon rank sum test). The incubation time before the addition of brefeldin A for detecting both MIG and IFN-γ responses by ICS, was determined to be 6 h.

As brefeldin A may be toxic to cells (Pommepuy et al., 2003), an experiment was carried out to determine the optimum time of incubation after adding brefeldin A. Cells from 5 normal human donors were incubated for 6 h with CEF or PPD, then for a further 6 h or 18 h with brefeldin A. Both MIG and IFN-γ production were analysed (data not shown). No significant difference was seen between the two incubation times (Student's paired *t*-test) in either cytokine.

### 3.3 Effect of co-stimulatory antibodies on the detection of MIG

**Fig. 3:**
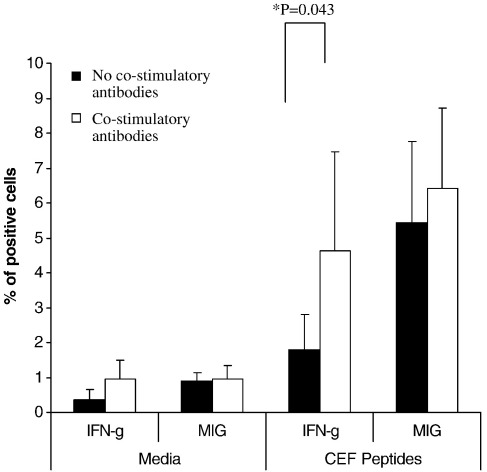
Experiment to test whether the addition of co-stimulatory antibodies, anti-CD28 and anti-CD49d, would increase the sensitivity of the MIG ICS assay. PBMC from 6 normal human donors were stimulated with CEF peptides both with and without the addition of co-stimulatory antibodies. Both MIG and IFN-γ production were measured. The data is presented as the mean and standard error of the percentage of IFN-γ positive cells within the CD3+ 7AAD-ve gate, or the mean and standard error of the percentage of MIG positive cells within the CD14+ 7AAD-ve gate. No significant differences in MIG secretion were seen in either CEF stimulated or media alone, between the samples with and without co-stimulatory antibodies (Student’s paired T-test). A significant difference in IFN-γ production was detected in the CEF stimulated samples between those with and without the co stimulatory antibodies (P= 0.043 Wilcoxon’s-Signed Rank test).

Co-stimulatory antibodies anti-CD28 and anti-CD49d have been shown to increase the sensitivity of the IFN-γ intracellular staining assay (Waldrop et al., 1998). An experiment to test the effect of co-stimulatory antibodies on the sensitivity of the MIG ICS and IFN-γ ICS assay was carried out using PBMC from 6 normal human donors. Cells were incubated with CEF and media alone, both with and without co-stimulatory antibodies. No significant differences (Student's paired *t*-test) in MIG production between the samples with co-stimulatory antibodies and the samples without co-stimulatory antibodies were seen (Fig. 3), however a significant difference in IFN-γ production was detected in the CEF stimulated samples between those with and without the co stimulatory antibodies (*P* *=* 0.043 Wilcoxon's-Signed Rank test).

The lack of increase in MIG secretion with the addition of co-stimulatory molecules may indicate that the co-stimulatory molecules do not enhance IFN-γ secretion from the cell, only intracellular IFN-γ production. In support of this theory, Jennes et al. showed that co-stimulatory molecules did not enhance the secretion of IFN-γ as detected by IFN-γ ELISPOT (Jennes et al., 2002).

### 3.4 Dose–response curve of MIG to recombinant human IFN-γ (rhIFN-γ)

**Fig. 4:**
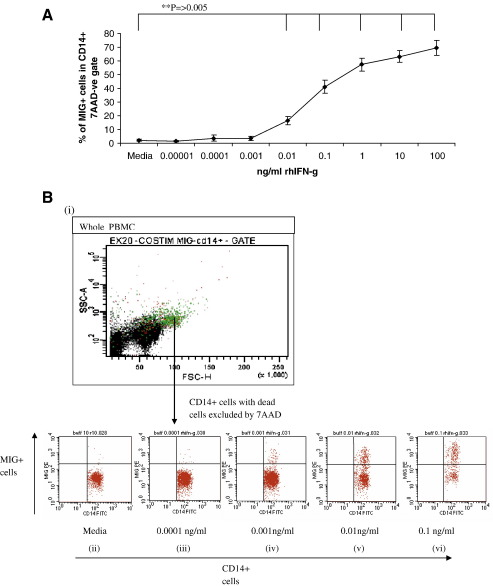
Investigation into the amount of rhIFN-γ needed to induce a MIG ICS response. Increasing concentrations of rhIFN-γ were added to PBMC from 6 donors. The cells were then incubated for 6 hours before and after the addition of brefeldin A. The data are presented as the mean percentage of MIG+ cells within the CD14+ population ± standard deviation (SD) (fig. 4A). A significant difference in MIG secretion was seen between the media and 0.01 ng/ml rhIFN-γ (P=0.005 -Student’s paired T-test), and all the higher concentrations of rhIFN-γ. Dot plots of one volunteer’s MIG responses to rhIFN-γ are shown in figure 4B. Firstly dead cells were excluded with 7AAD dead cell marker. Secondly the CD14+ cells were then selected against forward scatter. The CD14+ population is shown in green in plot (i). The following plots (ii-vi) show the MIG production within the CD14+ population. Responses to media (ii), 0.0001 ng/ml rhIFN-γ (iii), 0.001 ng/ml rhIFN-γ (iv), 0.01 ng/ml rhIFN-γ (v) and 0.1 ng/ml rhIFN-γ (vi) are shown.

It has been shown previously that MIG is induced by IFN-γ (Farber, 1990, 1993; Amichay et al., 1996). PBMC from 6 different donors were stimulated with increasing concentrations of rhIFN-γ. Stimulation with rhIFN-γ induced MIG expression in a dose dependent manner (Fig. 4A and B). A significant number of MIG secreting cells can be detected above background from as little as 0.01 ng/ml rhIFN-γ. The standard deviation between the 6 individuals tested was small and the ability of CD14+ cells to respond to IFN-γ in PBMC cell culture is reproducible between individuals.

### 3.5 Comparison of MIG ICS and IFN-γ intracellular staining assays in volunteers vaccinated with a malaria vaccine

**Fig. 5:**
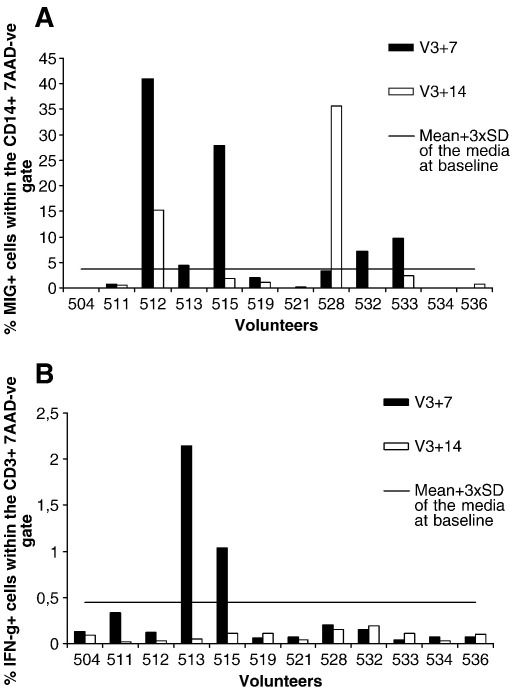
(A) shows the percentage of MIG+ cells within the CD14+7AAD-ve gate in volunteers vaccinated with the PFPFPM vaccine regimen (n=12). V3+7 are shown in black and V3+14 are shown in white. The line indicates the cut of for positivity, determined by 3 standard deviations above the mean of the media wells at the baseline time point. Five of 12 positive responses detected by the MIG ICS assay at V3+7 and 2/12 positive responses at V3+14. (B) shows the percentage of IFN-γ+ cells within CD3+ 7AAD-ve gate in volunteers vaccinated with the PFPFPM vaccine regimen. The responses at the V3+7 time point are shown in black, and V3+14 are shown in white. The line indicates the cut of for positivity, determined by 3 standard deviations above the mean of the media wells at the baseline time point. Two of 12 of the volunteers induced a positive IFN-γ ICS response at the V3+7 time point. The % of MIG+ cells detected by ICS above the media is significantly higher than the % of IFN-γ + cells detected by ICS above the media control at these time points (Wilcoxon Signed Ranks Test P=0.00039).

The optimised MIG ICS assay was then performed using PBMC from 12 volunteers vaccinated with the PFPFPM vaccine regimen. PBMC taken before the vaccination (D0), 7 days after the final vaccination (V3 + 7) and 14 days after the final vaccination (V3 + 14) were stimulated with a pool of TRAP peptides for a total of 12 h. The percentage of MIG+ cells within the CD14+ population is shown in Fig. 5A. Fig. 5B shows the antigen induced IFN-γ responses detected by ICS. A positive response in both the MIG ICS and the IFN-γ ICS was defined as more than 3 standard deviations above the mean of media alone at baseline (D0). For the IFN-γ ICS this value was 0.451% IFN-γ+ CD3+ cells, and for the MIG ICS it was 3.7%MIG+CD14+ cells. The majority of the IFN-γ ICS responses fell below the limits of detection of the assay (2 volunteers were positive at V3 + 7 time point). Five positive responses were detected by the MIG ICS assay at V3 + 7 and 2 positive responses at V3 + 14. The percentage of MIG+ cells detected by ICS above the media was also significantly higher than the percentage of IFN-γ + cells detected by ICS above the media control at these time points (Wilcoxon Signed Rank Test *P* = 0.00039).

### 3.6 The *ex vivo* IFN-γ ELISPOT assay positively correlates with the MIG ICS and MIG RT-PCR assay

**Fig. 6:**
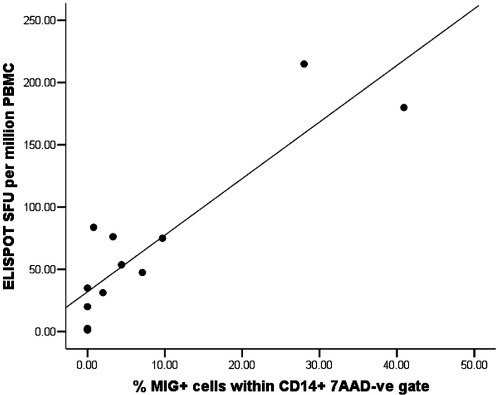
Shows that the ex vivo ELISPOT responses (SFU per million PBMC) significantly correlated with % of MIG+ cells within the CD14+ 7AAD-ve gate at V3+7 (Spearman’s correlation = 0.793, 2-tailed significance P=0.002) (n=12).

An *ex vivo* ELISPOT assay for IFN-γ was also carried out on these volunteers. Fig. 6 shows the positive correlation between the *ex vivo* ELISPOT responses and the MIG ICS responses at V3 + 7 (Spearman's correlation = 0.793, 2-tailed significance *P* = 0.002). The *ex vivo* IFN-γ ELISPOT also positively correlated with MIG RT-PCR at the V3 + 14 time point (Spearman's correlation = 0.661, 2-tailed significance *P* = 0.038) (data not shown). The MIG ICS assay did not correlate with the IFN-γ ICS assay, and the MIG RT-PCR assay did not correlate with the IFN-γ RT-PCR assay. This may be due to the low level responses seen in the IFN-γ ICS and IFN-γ RT-PCR assays.

## 4 Discussion

MIG is a chemokine produced largely by CD14+ monocytes and macrophages, and is induced via the JAK-STAT pathway by engagement of IFN-γ with it's receptor. It has not been shown that antigen internalisation by CD14+ cells causes MIG secretion directly, although this remains to be tested. Detection of MIG is therefore thought to be an indication of biologically active IFN-γ, a functional IFN-γR and JAK1-STAT1 pathway. In this paper we have shown that detecting MIG secretion by CD14+ cells using flow cytometry can be a sensitive method for measuring immune responses induced by vaccination, and that MIG detection by flow cytometry and RT-PCR can be more sensitive than detecting IFN-γ with these methods.

We found that rhIFN-γ can induce MIG secretion by CD14+ cells in a dose dependent manner. The standard deviation between the individuals tested was low which suggests that the measurement of MIG by ICS may be a useful way to estimate the amount of bioactive IFN-γ present in a culture.

A MIG ICS response significantly above the background could be detected when 0.01 ng/ml rhIFN-γ is added. In contrast, the cut off for sensitivity used to detect IFN-γ by ELISA in most reports is 0.1 ng/ml IFN-γ (Brahmbhatt et al., 2002; Black et al., 2003; Pinder et al., 2004). PBMC from naturally exposed volunteers stimulated with TRAP antigens have been reported to induce above 0.02 ng/ml (John et al., 2004), the amount of rhIFN-g shown to induce MIG is therefore within the range seen in TRAP stimulated PBMC. The MIG ICS assay may therefore be useful as a more sensitive method for detecting IFN-γ secretion.

MIG production has previously been reported in response to CMV, EBV and the circumsporozoite protein (CS) of *P. falciparum* in healthy volunteers*,* as well as CFP10 and ESAT6 in tuberculosis patients (Brice et al., 2001; Abramo et al., 2006). Both of these studies showed MIG detection to be a sensitive measure of immunogenicity. The sensitivity of the MIG ICS compared to the IFN-γ ICS was tested here in volunteers vaccinated with three PEV3A vaccinations in combination with the FFM vaccine regimen encoding ME-TRAP (PFPFPM). More positive responses were detected when MIG was analysed with ICS, than when IFN-γ was analysed by ICS. This indicates that MIG may be expressed and secreted at a higher level than IFN-γ. The detection of MIG by ICS might therefore have a lower threshold of sensitivity than the IFN-γ ICS assay. MIG production was only induced in the antigen stimulated samples, not the non-stimulated samples. This suggests that the MIG production was antigen specific and probably induce by IFN-γ originating from T-cells. It is however possible that the IFN-γ may have been supplied by cells other than T-cells such as NK or gammadelta T cells. Studies are currently being carried out to investigate alternative sources for the IFN-γ.

IFN-α can also induce MIG expression (Mahalingam et al., 2001). IFN-α RT-PCR was also carried out on the PFPFPM vaccinated volunteers, no significant difference was seen in IFN-α expression between D0 and V3 + 7 indicating that the MIG secretion was induced by IFN-γ.

As well as the potential sensitivity of the MIG ICS assay, the detection of MIG eliminates the need for the use of expensive co-stimulatory antibodies. Secondly, the MIG ICS assay can be performed using frozen cells, which is useful for large-scale vaccine trials where many of the biological samples are frozen down. Thirdly as the population of MIG secreting CD14+ cells is large, fewer cells are needed for the detection of a statistically significant response.

In these experiments, both the MIG ICS assay and MIG RT-PCR assays were shown to positively correlate with the *ex vivo* IFN-γ ELISPOT. A previous report analysing MIG secretion in response to *M. tuberculosis* specific antigens in tuberculosis patients and BCG vaccinated volunteers also showed MIG secretion to correlate with the *ex vivo* IFN-γ ELISPOT (Abramo et al., 2006).

Many approaches are being investigated with the aim of identifying a sensitive method for immuno-monitoring in vaccine trials, and vaccine trials on infants need to identify immune responses in small volumes of blood. Using MIG as a marker for antigen specific inflammatory immune responses may increase sensitivity without the need for increasing the volume of blood needed. The data presented here suggest that ICS measurement of MIG could form a useful addition to current methods used to detect vaccine induced T-cells.
