# Corrigendum to “MIG (CXCL9) is a more sensitive measure than IFN-γ of vaccine induced T-cell responses in volunteers receiving investigated malaria vaccines” [J. Immunol. Methods 340 (2009) 33–41]

**DOI:** 10.1016/j.jim.2011.03.002

**Published:** 2011-05-31

**Authors:** Tamara K. Berthoud, Susanna J. Dunachie, Stephen Todryk, Adrian V.S. Hill, Helen A. Fletcher

**Affiliations:** a University of Oxford, Centre for Clinical Vaccinology and Tropical Medicine, Churchill Hospital, Oxford, OX3 7LJ, UK; b School of Applied Sciences, Northumbria University, Newcastle-upon-Tyne, UK

The authors regret the following acknowledgment was missing from the original publication of this article. The acknowledgment has been reproduced below:

The study was supported by funding from the NIHR Oxford Biomedical Research Centre programme.

